# The Independent Prognostic Effect of Lymph Node Dissection on Patients With Stage IA NSCLC With Different T Stages

**DOI:** 10.3389/fsurg.2021.798046

**Published:** 2021-12-10

**Authors:** Dechang Zhao, Rusi Zhang, Longjun Yang, Zirui Huang, Yongbin Lin, Yingsheng Wen, Xuewen Zhang, Gongming Wang, Guangran Guo, Xiangyang Yu, Weidong Wang, Kexing Xi, Lanjun Zhang

**Affiliations:** ^1^State Key Laboratory of Oncology in South China, Collaborative Innovation Center for Cancer Medicine, Guangzhou, China; ^2^Department of Thoracic Surgery, Sun Yat-sen University Cancer Center, Guangzhou, China; ^3^Department of Anesthesiology, Sun Yat-sen University Cancer Center, Guangzhou, China; ^4^Department of Thoracic Surgical Oncology, National Cancer Center/National Clinical Research Center for Cancer/Cancer Hospital, Chinese Academy of Medical Sciences and Peking Union Medical College, Beijing, China; ^5^Department of Thoracic Surgery, The First Affiliated Hospital, School of Medicine, Zhejiang University, Hangzhou, China; ^6^Department of Colorectal Surgery, National Cancer Center/National Clinical Research Center for Cancer/Cancer Hospital, Chinese Academy of Medical Sciences and Peking Union Medical College, Beijing, China

**Keywords:** non-small cell lung cancer (NSCLC), lymph node dissection, prognosis, X-tile software, surveillance, epidemiology and end results (SEER) database

## Abstract

**Background:** Currently, the extent of lymph node evaluation necessary for patients with early-stage non-small-cell lung cancer (NSCLC) remains controversial according to the latest ESMO and NCCN guidelines. In this study, we aimed to evaluate the survival effect of different numbers of lymph nodes examined (LNE) and regions of lymph nodes removed (LNR) in patients with stage IA NSCLC.

**Method:** All patients with stage IA NSCLC undergoing lobectomy or bilobectomy were selected from the surveillance, epidemiology, and end results (SEER) database. The number of LNE and LNR were stratified into 4 groups (0, 1–2, 3–8, and ≥9 lymph nodes) and 3 groups (0, 1–3, and ≥4 regions) respectively. Additionally, the survival curves of overall survival (OS) and cancer-specific survival (CSS) were plotted and compared with the Kaplan-Meier method and log-rank test. Independent prognostic clinicopathological factors were evaluated via Cox proportional hazard regression and subgroup analysis.

**Results:** Totally, 12,490 patients with stage IA NSCLC were enrolled in our study. Patients with ≥9 LNE and ≥4 LNR in both the T1b and T1c stages consistently demonstrated the significantly best OS and CSS outcomes. In the multivariate analysis, patients with ≥9 LNE consistently had a significantly better CSS [hazards ration (HR) (95% CI):0.539 (0.438–0.663)], and those with ≥4 LNR consistently had a significantly better OS [HR (95% CI):0.678 (0.476–0.966)]. Furthermore, ≥9 LNE and ≥4 LNR were associated with better survival in most subgroups.

**Conclusion:** This study demonstrated that ≥9 LNE and ≥4 LNR are highly recommended for stage IA2 and stage IA3 patients but optional for stage IA1 patients.

## Introduction

Lung cancer is currently one of the most common and deadliest cancers in the world (1). Non-small-cell lung cancer (NSCLC) is the most common subtype and accounts for almost 85% of all lung cancer cases (2). Currently, the AJCC eighth edition TNM stage has been the basis for the choice of NSCLC treatment. According to the ESMO and NCCN guidelines for NSCLC, lobectomy is still the standard treatment for stage IA NSCLC. However, for the management of lymph nodes during surgery, the choice between systematic lymphadenectomy (LA) and lymph node sampling (LS) remains unclear (3, 4). The International Association for the Study of Lung Cancer (IASLC) defined systematic nodal dissection, which had excision of ≥6 lymph nodes and ≥3 nodal stations, including the subcarinal station (5).

In a prospective study during the 1990s, the difference in survival benefit between LA and LS was not observed in NSCLC patients with pN0 (6). Additionally, the same conclusion was supported by the results of the American College of Surgery Oncology Group (ACOSOG) Z0030 Trial (7). However, another previous study during the 2000s oppositely confirmed that LA was associated with better survival than LS in stage I NSCLC patients (8). The ESTS guidelines in 2006 also recommended LA in NSCLC patients (9). Beyond that, the positive influence of more lymph nodes sampled on survival in stage I NSCLC patients was confirmed (10, 11). Therefore, the prognostic effects of the number of lymph nodes examined (LNE) and scope of regional lymph nodes removed (LNR) in patients with stage IA NSCLC are still unclear and need to be solved.

In this study, we performed a retrospective population-based analysis of the surveillance, epidemiology, and end results (SEER) cancer database and aimed to assess the prognostic effect of LNE and LNR in patients with stage IA NSCLC who underwent anatomic pulmonary resection. Moreover, we used the AJCC eighth edition TNM stage as the basis for staging NSCLC in our study, which has not been used in previous studies.

## Methods

### Patient Selection

The SEER database is funded by the Nation Cancer Institute and covers approximately 28% of the United States population (12). Therefore, it is a comprehensive and representative source of demographic, clinicopathological, and survival information from many kinds of cancer patients. According to the guidelines of the SEER database, permission to use the data was obtained (reference number 14,683-Nov2019). For the further analysis of the T stage, we needed to divide the T stage into three groups (T1a, T1b, and T1c) according to the eighth edition (AJCC) American Joint Committee on Cancer TMN stage. For the SEER database, the latest T stage classification can only be inferred from the variable “CS TUMOR SIZE (2004–2015),” which is only available for patients diagnosed between 2004 and 2015. Therefore, all patients with stage IA NSCLC diagnosed between 2004 and 2015 were selected from the SEER database.

The specific inclusion criteria were as follows: (1) patients diagnosed with only one primary lung cancer (ICD-O-3 primary site codes: C340-343 and C348-349); (2) patients diagnosed with NSCLC (ICD-O-3 histology code: large cell carcinoma 8012–8014; adenocarcinoma 8140–8147, 8250–8255, 8310, 8333, 8470, 8480, 8481, 8490, 8550, and 8551; squamous cell carcinoma 8052, 8070–8078, and 8083; and adenosquamous cell carcinoma 8560); (3) patients diagnosed with stage IA; (4) patients treated with lobectomy or bilobectomy; and (5) patients with positive histological or immunophenotyping diagnosis.

The exclusion criteria were as follows: (1) patients diagnosed with autopsy; (2) patients without complete demographic and clinicopathologic information; (3) patients without a record of chemotherapy and radiotherapy; (4) patients without complete survival states and time; (5) patients without the scope of lymph nodes removed and the number of lymph nodes examined; (6) patients with a follow-up time of <1 month.

### Statistical Analysis

In our study, we used the National Cancer Institute's SEER^*^Stat software [version 8.3.6; SEER 18 Regs Custom Data (with additional treatment fields), November 2018 Sub (1975–2016 varying) database]. Age, gender, race, histology, grade, T stage (AJCC eighth edition), number of LNE, the scope of regional LNR, chemotherapy, and radiotherapy were included as possible confounding factors. Additionally, overall survival (OS) and cancer-specific survival (CSS) were used as prognostic indicators. Before the statistical analyses, stratified cut-off points of the number of lymph nodes examined were determined by using X-tile software (version 3.6.1) (13). Consequently, the number of lymph nodes examined was classified into four groups (0, 1–2, 3–8, and ≥9). Additionally, the LNR has been classified into 3 groups (0, 1–3, and ≥4 regions) which was set up by the SEER database. All related demographic and clinicopathological characteristics are presented as numbers and percentages. Associations between T stage groups and demographic and clinicopathological characteristics were analyzed by Pearson's chi-square test, which was similarly used to identify the correlation between LNE and LNR. Survival curves were generated by the Kaplan-Meier method and compared by log-rank tests. Furthermore, Cox proportional hazards ratio regression models were performed to assess the influence of all variables on OS and CSS by using forward stepwise methods for both univariate and multivariate analysis.

A two-tailed *p*-value < 0.05 was considered statistically significant. All statistical analysis was conducted using SPSS (version 26.0; IBM Corporation, Armonk, NY, USA) and R (version 3.6.3; R Development Core Team, http://www.r-project.org).

## Results

### Demographic and Clinicopathological Characteristics of the Patients

A total of 12,490 patients with stage IA NSCLC were enrolled in our study, among which 1,074 (8.6%), 6,353 (50.9%), and 5,063 (40.5%) patients were diagnosed with T1a, T1b, and T1c disease, respectively. Detailed information about demographic and clinicopathological characteristics is shown in Table 1 (stratified by T stage). The chi-square test confirmed that patients with different T stages had significant differences in age (*P* < 0.001), gender (*P* < 0.001), race (*P* = 0.048), histology (*P* < 0.001), grade (*P* < 0.001), chemotherapy (*P* < 0.001), and radiotherapy (*P* = 0.001). However, there was no significant difference in either the number of LNE (*P* = 0.357) or the scope of regional LNR (*P* = 0.376). Furthermore, there was a significant correlation between the number of LNE and the scope of regional LNR, with *P* < 0.001 and Pearson's *R* = 0.698 (Table 2).

**Table 1:** Clinicopathological characteristics of NSCLC patients in the SEER database.

**Variable**	**Total**	**T1a**	**T1b**	**T1c**	***P* value**
	**(*n* = 12,490)**	**(*n* = 1,074, 8.6%)**	**(*n* = 6,353, 50.9 %)**	**(*n* = 5,063, 40.5%)**	
**Age**					P < 0.001
<70	7,281 (58.3%)	705 (65.6%)	3,867 (60.9%)	2,709 (53.5%)	
≥70	5,209 (41.7%)	369 (34.4%)	2,486 (39.1%)	2,354 (46.5%)	
**Gender**					*P* < 0.001
Male	5,276 (42.2%)	384 (35.8%)	2,614 (41.1%)	2,278 (45.0%)	
Female	7,214 (57.8%)	690 (64.2%)	3,739 (58.9%)	2,785 (55.0%)	
**Race**					*P* = 0.048
White	10,490 (84.0%)	929 (86.5%)	5,350 (84.2%)	4,211 (83.2%)	
Black	1,031 (8.3%)	83 (7.7%)	510 (8.0%)	438 (8.7%)	
Other	969 (7.8%)	62 (5.8%)	493 (7.8%)	414 (8.2%)	
**Histology**					*P* < 0.001
ADC	9,046 (72.4%)	821 (76.4%)	4,767 (75.0%)	3,458 (68.3%)	
SCC	2,925 (23.4%)	224 (20.9%)	1,347 (21.2%)	1,354 (26.7%)	
LCC	265 (2.1%)	20 (1.9%)	114 (1.8%)	131 (2.6%)	
ASC	254 (2.0%)	9 (0.8%)	125 (2.0%)	120 (2.4%)	
**Grade**					P < 0.001
Well differentiated	2,925 (23.4%)	388 (36.1%)	1,534 (24.1%)	1,003 (19.8%)	
Moderately differentiated	6,104 (48.9%)	461 (42.9%)	3,176 (50.0%)	2,467 (48.7%)	
Poorly differentiated	3,316 (26.5%)	219 (20.4%)	1,582 (24.9%)	1,515 (29.9%)	
Undifferentiated	145 (1.2%)	6 (0.6%)	61 (1.0%)	78 (1.5%)	
**The number of lymph nodes examined**					*P* = 0.357
0	376 (3.0%)	38 (3.5%)	190 (3.0%)	148 (2.9%)	
1–2	1,223 (9.8%)	107 (10.0%)	647 (10.2%)	469 (9.3%)	
3–8	5,628 (45.1%)	477 (44.4%)	2,892 (45.5%)	2,259 (44.6%)	
≥9	5,263 (42.1%)	452 (42.1%)	2,624 (41.3%)	2,187 (43.2%)	
**The scope of regional lymph nodes removed**					*P* = 0.376
0 region	349 (2.8%)	36 (3.4%)	183 (2.9%)	130 (2.6%)	
1–3 regions	2,194 (17.6%)	203 (18.9%)	1,115 (17.6%)	876 (17.3%)	
≥4 regions	9,947 (79.6%)	835 (77.7%)	5,055 (79.6%)	4,057 (80.1%)	
**Chemotherapy**					P < 0.001
No	12,055 (96.5%)	1,046 (97.4%)	6,177 (97.2%)	4,832 (95.4%)	
Yes	435 (3.5%)	28 (2.6%)	176 (2.8%)	231 (4.6%)	
**Radiotherapy**					*P* = 0.001
No	12,270 (98.2)	1,059 (98.6%)	6,265 (98.6%)	4,946 (97.7%)	
Yes	220 (1.8%)	15 (1.4%)	88 (1.4%)	117 (2.3%)	

*ADC, Adenocarcinoma; SCC, Squamous cell carcinoma; LCC, Large Cell Carcinoma; ASC, Adenosquamous carcinoma*.

**Table 2:** The correlation between the number of lymph nodes examined and the scope of regional lymph nodes removed.

**Variable**	**Total**	**The scope of regional lymph nodes removed**			***P* value**
		**0 region**	**1–3 regions**	**≥4 regions**	
	**(*n* = 12,490)**	**(*n* = 349, 2.8%)**	**(*n* = 2,194, 17.6 %)**	**(*n* = 9,947, 79.6%)**	
**The number of lymph nodes examined**					*P* < 0.001
0	376 (3.0%)	310 (88.8%)	14 (0.6%)	52 (0.5%)	
1–2	1,223 (9.8%)	8 (2.3%)	1,185 (54.0%)	30 (0.3%)	
3–8	5,628 (45.1%)	22 (6.3%)	965 (44.0%)	4,641 (46.7%)	
≥9	5,263 (42.1%)	9 (2.6%)	30 (1.4%)	5,224 (52.5%)	

### Survival Analysis

**Figure 1:**
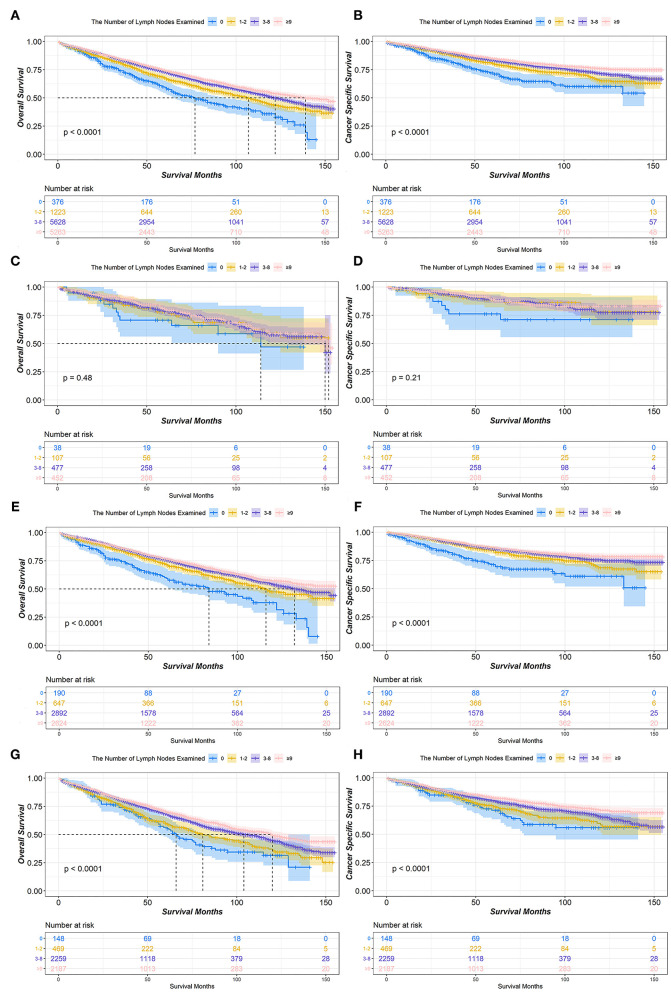
Kaplan-Meier survival curves of the number of lymph nodes examined in IA non-small cell lung cancer (NSCLC) patients who underwent lobectomy or bilobectomy. Overall survival comparison among 0, 1–2, 3–8, and ≥9 lymph nodes examined in stage IA **(A)** NSCLC patients including T1a **(C)**, T1b **(E)**, and T1c **(G)**. Cancer-specific survival comparison among 0, 1–2, 3–8, and ≥9 lymph nodes examined in stage IA **(B)** NSCLC patients including T1a **(D)**, T1b **(F)**, and T1c **(H)**.

The median OS for patients with stage IA NSCLC who underwent lobectomy or bilobectomy according to the number of LNE was 77 months for 0 LNE, 107 months for 1–2 LNE, 122 months for 3–8 LNE, and 139 months for ≥9 LNE. Moreover, the difference in OS was significant (*P* < 0.001). Although the median CSS in all groups of the number of LNE was not reached, the difference in CSS was still significant (*P* < 0.001). In the T1a stage, there was no significant difference in either OS (*P* = 0.48) or CSS (*P* = 0.21). However, in the T1b and T1c stages, the ≥9 LNE group had significantly better OS and CSS (Figure 1).

**Figure 2:**
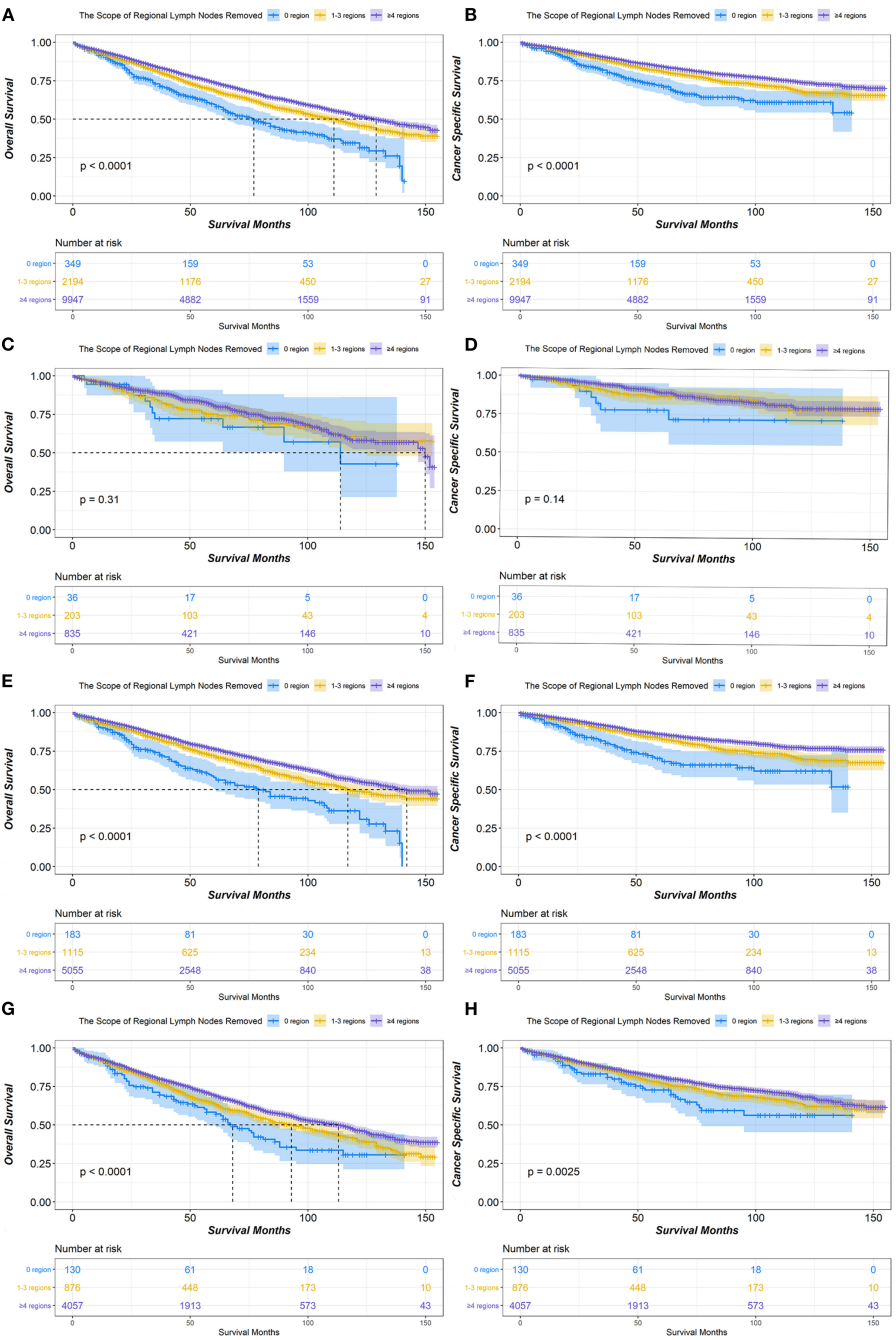
Kaplan-Meier survival curves of the scope of regional lymph nodes removed in IA non-small cell lung cancer (NSCLC) patients who underwent lobectomy or bilobectomy. Overall survival comparison among 0, 1–3, and ≥4 regions of lymph nodes removed in stage IA **(A)** NSCLC patients including T1a **(C)**, T1b **(E)**, and T1c **(G)**. Cancer-specific survival comparison among 0, 1–3, and ≥4 regions of lymph nodes removed in stage IA **(B)** NSCLC patients including T1a **(D)**, T1b **(F)**, and T1c **(H)**.

In the survival analysis of the scope of regional LNR, the median OS for patients with stage IA NSCLC treated with lobectomy or bilobectomy was 77 months for 0 LNR, 111 months for 1–3 LNR, and 129 months for ≥4 LNR. Additionally, a significant difference in OS was observed (*P* < 0.001). The median CSS in all groups of the scope of regional LNR was not reached, but the ≥4 LNR group had significantly better CSS than both the 0 and 1–3 LNR groups (*P* < 0.001). The ≥4 LNR group consistently demonstrated the best OS and CSS outcomes among the different scope of regional LNR groups in patients with T1b or T1c stage disease. However, for patients with T1a stage disease, there was no significant difference among 0, 1–3, and ≥4 LNR in either OS (*P* = 0.31) or CSS (*P* = 0.14) (Figure 2).

### Cox Proportional Hazards Regression Model

To further explore whether the different number of LNE and scope of regional LNR had different effects on patients with stage IA NSCLC in terms of OS and CSS, we performed an analysis of the related variables via a Cox proportional hazards regression model. In the univariate analysis, with 0 LNE as a reference, patients with ≥9 LNE had significantly better OS [hazards ratio [HR] (95% CI):0.552 (0.472–0.644), *P* < 0.001] and CSS [HR (95% CI):0.496 (0.404–0.61), *P* < 0.001] than those with 1–2 LNE [OS: HR (95% CI):0.724 (0.61–0.858), *P* < 0.001; CSS: HR (95% CI):0.681.542–0.856), *P* = 0.001] and 3–8 LNE [OS: HR (95% CI):0.606 (0.52–0.706), *P* < 0.001; CSS: HR (95% CI):0.572 (0.467–0.701), *P* < 0.001]. Additionally, patients with ≥4 LNR had significantly better OS [HR (95% CI):0.565 (0.484–0.661), *P* < 0.001) and CSS (HR (95% CI):0.526 (0.427–0.647), *P* < 0.001] than those with 1–3 LNR [OS: HR (95% CI):0.681 (0.577–0.803), *P* < 0.001; CSS: HR (95% CI):0.648 (0.519–0.808), *P* < 0.001], with 0 LNR as a reference. Furthermore, in the multivariate Cox proportional hazards regression analysis, patients with ≥4 LNR had significantly better OS [HR (95% CI):0.678 (0.476–0.966), *P* = 0.311], and those with 1–2, 3–8, and ≥9 LNE consistently had significantly better CSS than those with 0 LNE [1–2 LNE: HR (95% CI):0.734 (0.583–0.923), *P* = 0.008; 3–8 LNE: HR (95% CI):0.627 (0.511–0.769), *P* < 0.001; ≥9 LNE: HR (95% CI):0.539 (0.438–0.663), *P* < 0.001] (Table 3). However, when the multivariate analysis was performed without LNE enrollment, patients with ≥4 LNR had a significantly lower HR in both OS and CSS (Table 4). Consistently, patients with ≥9 LNE had significantly better OS and CSS according to the multivariate analysis without LNR enrollment (Table 5).

**Table 3:** Univariate and multivariate analysis of overall survival and cancer-specific survival.

	**Overall survival**				**Cancer specific survival**			
**Variable**	**Univariate**		**Multivariate**		**Univariate**		**Multivariate**	
	**Hazard ratio (95%CI)**	***P* value**	**Hazard ratio (95%CI)**	***P* value**	**Hazard ratio (95%CI)**	***P* value**	**Hazard ratio (95%CI)**	***P* value**
**Age**								
<70	Reference		Reference		Reference		Reference
≥70	1.907 (1.790–2.031)	*p* < 0.001	1.883 (1.766–2.007)	*p* < 0.001	1.479 (1.373–1.633)	*p* < 0.001	1.524 (1.396–1.665)	*p* < 0.001
**Gender**								
Male	Reference		Reference		Reference		Reference
Female	0.650 (0.610−0.692)	*p* < 0.001	0.702 (0.659–0.748)	*p* < 0.001	0.716 (0.657–0.781)	*p* < 0.001	0.777 (0.712–0.849)	*p* < 0.001
**Race**								
White	Reference		Reference		Reference		Reference
Black	1.008 (0.898–1.131)	*P* = 0.898	1.060 (0.944–1.191)	*P* = 0.325	1.166 (1.004–1.354)	*P* = 0.044	1.165 (1.002–1.354)	*P* = 0.047
Other	0.575 (0.496–0.668)	*p* < 0.001	0.635 (0.547–0.738)	*p* < 0.001	0.639 (0.524–0.779)	*p* < 0.001	0.699 (0.573–0.852)	*p* < 0.001
**Histology**								
ADC	Reference		Reference		Reference		Reference
SCC	1.724 (1.609–1.846)	*p* < 0.001	1.278 (1.189–1.375)	*p* < 0.001	1.387 (1.256–1.531)	*p* < 0.001	1.017 (0.917–1.128)	*P* = 0.746
LCC	2.016 (1.701–2.389)	*p* < 0.001	1.562 (1.281–1.905)	*p* < 0.001	2.199 (1.766–2.737)	*p* < 0.001	1.468 (1.134–1.899)	*P* = 0.004
ASC	1.584 (1.294–1.938)	*p* < 0.001	1.203 (0.981–1.474)	*P* = 0.076	1.405 (1.057–1.869)	*P* = 0.019	1.021 (0.766–1.360)	*P* = 0.888
**Grade**								
Well differentiated	Reference		Reference		Reference		Reference
Moderately differentiated	1.856 (1.687–2.042)	*p* < 0.001	1.635 (1.482–1.803)	*p* < 0.001	2.127 (1.850–2.446)	*p* < 0.001	1.986 (1.723–2.290)	*p* < 0.001
Poorly differentiated	2.405 (2.176–2.657)	*p* < 0.001	1.931 (1.736–2.148)	*P* < 0.001	2.930 (2.537–3.385)	*p* < 0.001	2.526 (2.170–2.941)	*p* < 0.001
Undifferentiated	2.390 (1.841–3.103)	*p* < 0.001	1.496 (1.108–2.019)	*P* = 0.009	3.144 (2.225–4.443)	*p* < 0.001	1.930 (1.296–2.876)	*P* = 0.001
**T stage**								
1a	Reference		Reference		Reference		Reference
1b	1.221 (1.071–1.393)	*P* = 0.003	1.174 (1.029–1.339)	*P* = 0.017	1.319 (1.089–1.596)	*P* = 0.005	1.254 (1.036–1.518)	*P* = 0.020
1c	1.693 (1.437–1.869)	*p* < 0.001	1.416 (1.241–1.616)	*p* < 0.001	1.896 (1.568–2.293)	*p* < 0.001	1.638 (1.354–1.983)	*p* < 0.001
**The number of lymph nodes examined**								
0	Reference		Reference		Reference		Reference
1–2	0.724 (0.610–0.858)	*p* < 0.001	0.999 (0.700–1.425)	*P* = 0.996	0.681 (0.5420.856)	*P* = 0.001	0.734 (0.583–0.923)	*P* = 0.008
3–8	0.606 (0.520–0.706)	*p* < 0.001	0.910 (0.646–1.281)	*P* = 0.589	0.572 (0.467–0.701)	*p* < 0.001	0.627 (0.511–0.769)	*p* < 0.001
≥9	0.552 (0.472–0.644)	*p* < 0.001	0.829 (0.587–1.172)	*P* = 0.289	0.496 (0.404–0.610)	*p* < 0.001	0.539 (0.438–0.663)	*p* < 0.001
**The scope of lymph nodes removed**								
0 region	Reference		Reference		Reference		Reference
1–3 regions	0.681 (0.577–0.803)	*p* < 0.001	0.743 (0.520–1.062)	*P* = 0.103	0.648 (0.519–0.808)	*p* < 0.001		
≥4 regions	0.565 (0.484–0.661)	*p* < 0.001	0.678 (0.476–0.966)	*P* = 0.031	0.526 (0.427–0.647)	*p* < 0.001		
**Chemotherapy**								
No	Reference				Reference		Reference
Yes	1.385 (1.201–1.598)	*p* < 0.001			2.108 (1.787–2.487)	*p* < 0.001	1.392 (1.152–1.682)	*P* = 0.001
**Radiotherapy**								
No	Reference		Reference		Reference		Reference
Yes	2.764 (2.329–3.280)	*p* < 0.001	2.511 (2.113–2.985)	*p* < 0.001	4.232 (3.491–5.129)	*p* < 0.001	3.023 (2.428–3.763)	*p* < 0.001

*ADC, Adenocarcinoma; SCC, Squamous cell carcinoma; LCC, Large Cell Carcinoma; ASC, Adenosquamous carcinoma*.

**Table 4:** Multivariate analysis of overall survival and cancer-specific survival without the number of lymph nodes examined.

**Variable**	**Overall survival**		**Cancer specific survival**	
	**Hazard ratio (95%CI)**	***P* value**	**Hazard ratio (95%CI)**	***P* value**
**Age**				
<70	Reference		Reference
≥70	1.884 (1.767–2.008)	*p* < 0.001	1.526 (1.397–1.667)	*p* < 0.001
**Gender**				
Male	Reference		Reference
Female	0.701 (0.658–0.747)	*p* < 0.001	0.775 (0.710–0.846)	*p* < 0.001
**Race**				
White	Reference		Reference
Black	1.069 (0.952–1.200)	*P* = 0.261	1.178 (1.014–1.369)	*P* = 0.033
Other	0.636 (0.548–0.739)	*p* < 0.001	0.701 (0.575–0.856)	*p* < 0.001
**Histology**				
ADC	Reference		Reference
SCC	1.277 (1.187–1.373)	*p* < 0.001	1.017 (0.917–1.128)	*P* = 0.753
LCC	1.574 (1.291–1.919)	*p* < 0.001	1.507 (1.164–1.949)	*p* = 0.002
ASC	1.195 (0.975–1.465)	*P* = 0.086	1.003 (0.753–1.336)	*P* = 0.984
**Grade**				
Well differentiated	Reference		Reference
Moderately differentiated	1.636 (1.484–1.805)	<0.001	1.986 (1.722–2.289)	*p* < 0.001
Poorly differentiated	1.931 (1.736–2.148)	<0.001	2.521 (2.165–2.934)	*p* < 0.001
Undifferentiated	1.487 (1.102–2.006)	*P* = 0.010	1.880 (1.262–2.801)	*P* = 0.002
**T stage**				
1a	Reference		Reference
1b	1.177 (1.031–1.342)	*P* = 0.016	1.261 (1.041–1.526)	*P* = 0.018
1c	1.414 (1.293–1.614)	*p* < 0.001	1.642 (1.357–1.987)	*p* < 0.001
**The scope of lymph nodes removed**				
0 region	Reference		Reference
1–3 regions	0.717 (0.607–0.847)	*p* < 0.001	0.677 (0.542–0.846)	*P* = 0.001
≥4 regions	0.594 (0.508–0.695)	*p* < 0.001	0.552 (0.448–0.680)	*p* < 0.001
**Chemotherapy**				
No				Reference
Yes			1.394 (1.154–1.684)	*P* = 0.001
**Radiotherapy**				
No	Reference			Reference
Yes	2.525 (2.125–3.001)	*p* < 0.001	3.060 (2.458–3.809)	*p* < 0.001

*ADC, Adenocarcinoma; SCC, Squamous cell carcinoma; LCC, Large Cell Carcinoma; ASC, Adenosquamous carcinoma*.

**Table 5:** Multivariate analysis of overall survival and cancer-specific survival without the scope of lymph nodes removed.

**Variable**	**Overall survival**		**Cancer specific survival**	
	**Hazard ratio (95%CI)**	***P* value**	**Hazard ratio (95%CI)**	***P* value**
**Age**				
<70	Reference		Reference
≥70	1.881 (1.764–2.006)	*p* < 0.001	1.524 (1.396–1.665)	*p* < 0.001
**Gender**				
Male	Reference		Reference
Female	0.702 (0.659–0.749)	*p* < 0.001	0.777 (0.712–0.849)	*p* < 0.001
**Race**				
White	Reference		Reference
Black	1.059 (0.943–1.190)	*P* = 0.331	1.165 (1.002–1.354)	*P* = 0.047
Other	0.634 (0.546–0.737)	*p* < 0.001	0.699 (0.573–0.852)	*p* < 0.001
**Histology**				
ADC	Reference		Reference
SCC	1.277 (1.187–1.374)	*p* < 0.001	1.017 (0.917–1.128)	*P* = 0.746
LCC	1.540 (1.264–1.878)	*p* < 0.001	1.468 (1.134–1.899)	*p* = 0.004
ASC	1.211 (0.988–1.485)	*P* = 0.065	1.021 (0.766–1.360)	*P* = 0.888
**Grade**				
Well differentiated	Reference		Reference
Moderately differentiated	1.637 (1.485–1.806)	<0.001	1.986 (1.723–2.290)	*p* < 0.001
Poorly differentiated	1.933 (1.737–2.150)	<0.001	2.526 (2.170–2.941)	*p* < 0.001
Undifferentiated	1.519 (1.126–2.050)	*P* = 0.006	1.930 (1.296–2.876)	*P* = 0.001
**T stage**				
1a	Reference		Reference
1b	1.171 (1.026–1.336)	*P* = 0.019	1.254 (1.036–1.518)	*P* = 0.020
1c	1.412 (1.237–1.612)	*p* < 0.001	1.638 (1.354–1.983)	*p* < 0.001
**The number of lymph nodes examined**				
0	Reference		Reference
1–2	0.782 (0.659–0.928)	*P* = 0.005	0.734 (0.583–0.923)	*P* = 0.008
3–8	0.662 (0.567–0.772)	*p* < 0.001	0.627 (0.511–0.769)	*p* < 0.001
≥4	0.593 (0.508–0.693)	*p* < 0.001	0.539 (0.438–0.663)	*p* < 0.001
**Chemotherapy**				
No			Reference
Yes			1.392 (1.152–1.682)	*P* = 0.001
**Radiotherapy**				
No	Reference		Reference
Yes	2.495 (2.099–2.965)	*p* < 0.001	3.023 (2.428–3.763)	*p* < 0.001

*ADC, Adenocarcinoma; SCC, Squamous cell carcinoma; LCC, Large Cell Carcinoma; ASC, Adenosquamous carcinoma*.

**Figure 3:**
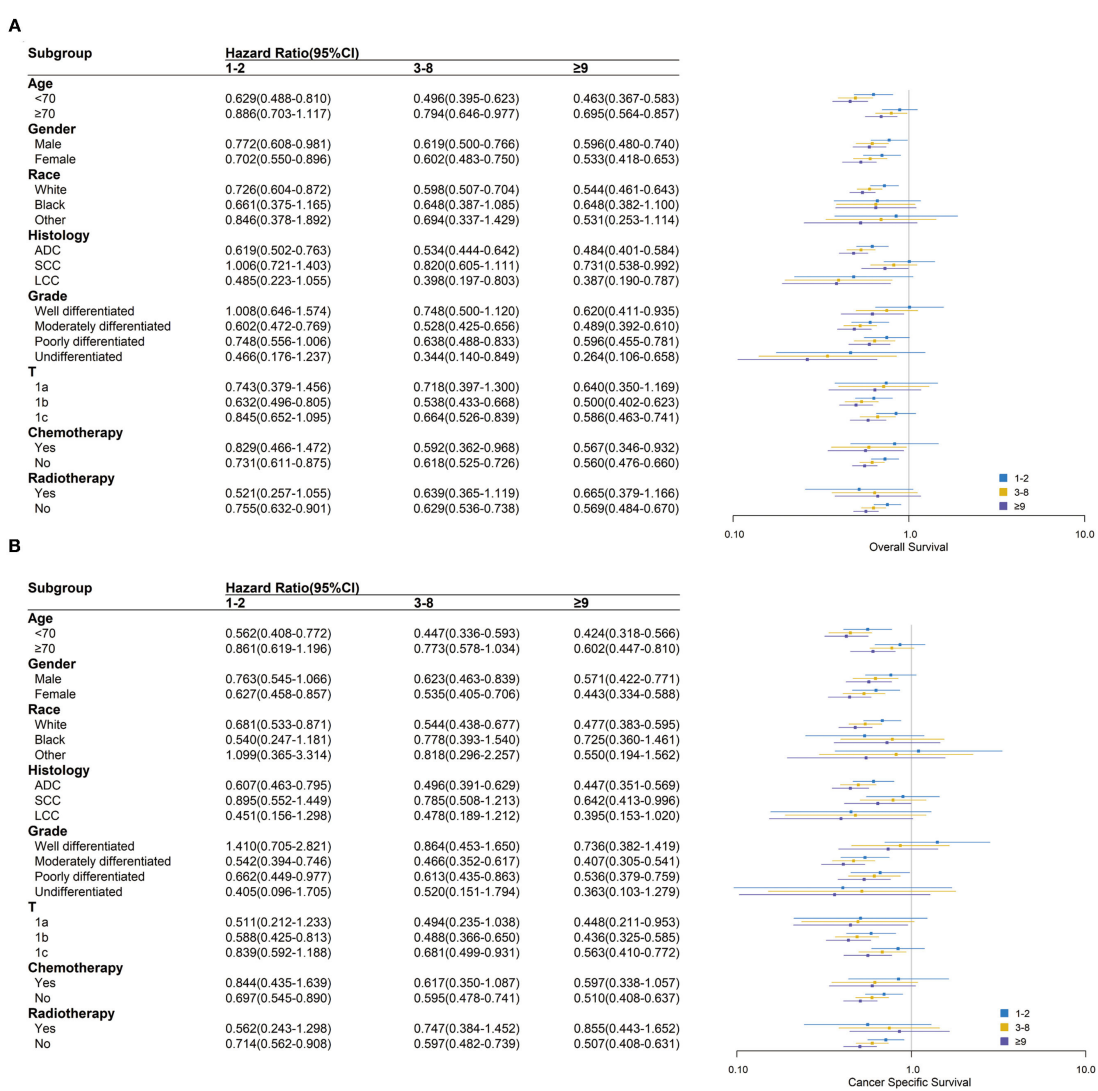
Over survival **(A)** and cancer-specific survival **(B)** comparison among 0, 1–2, 3–8, and ≥9 lymph nodes examined in different clinicopathological subgroups analysis. Blue, yellow and purple boxes represent the hazard ratios (HRs) of 1–2, 3–8 and ≥9 lymph nodes examined respectively. The lines represent the 95% CI of HR.

**Figure 4:**
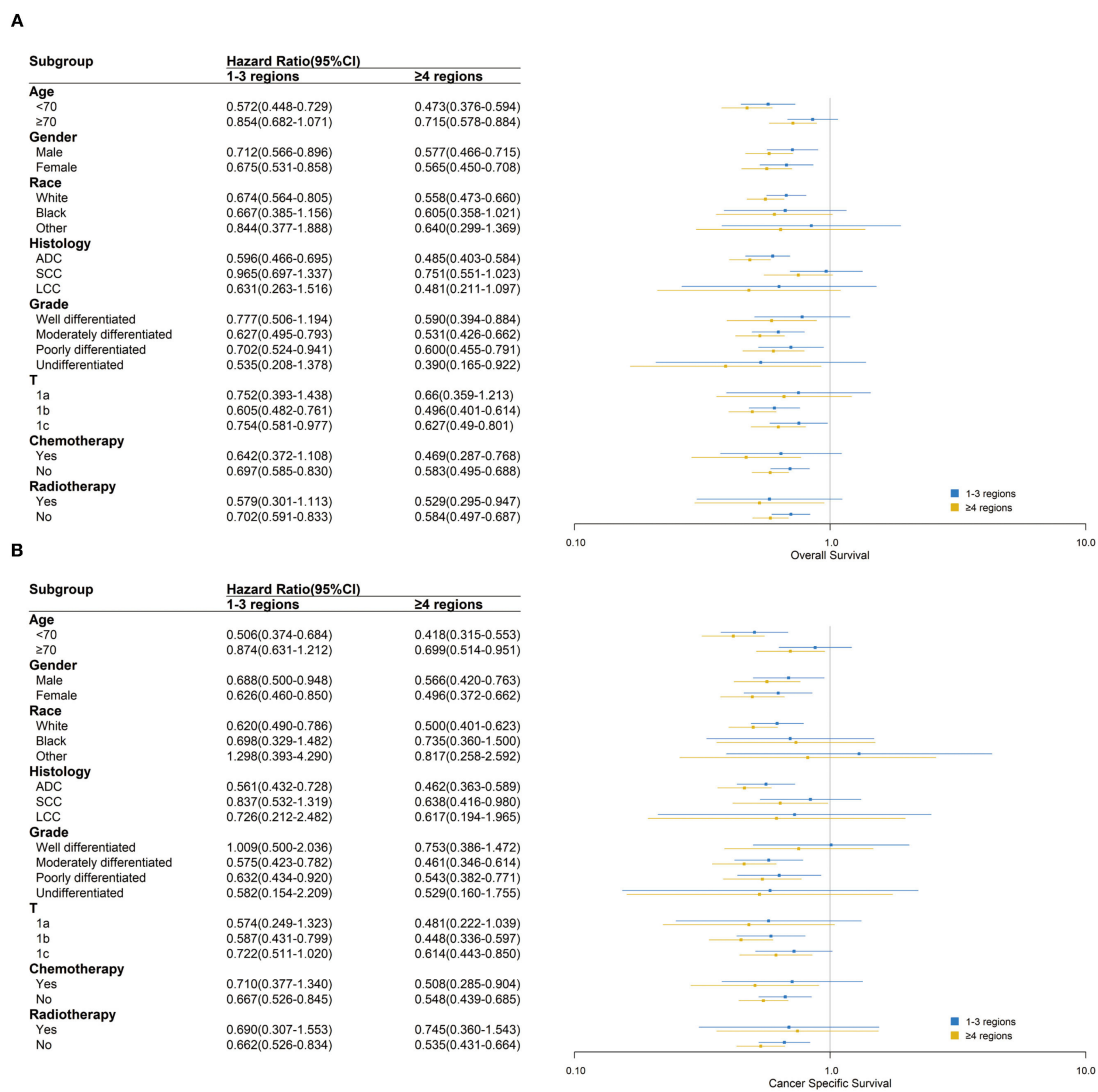
Over survival **(A)** and cancer-specific survival **(B)** comparison among 0, 1–3 and ≥4 regions lymph nodes removed in different clinicopathological subgroups analysis. Blue and yellow boxes represent the HRs of 1–3 and ≥4 regions lymph nodes removed, respectively. The lines represent the 95% CI of the hazard ratio.

In most subgroups of age, gender, race, histology, grade, T stage, chemotherapy, and radiotherapy, patients with ≥9 LNE had the lowest HR among all LNE subgroups for both OS and CSS, with 0 LNE as a reference. Therefore, the subgroup analysis consistently demonstrated that more LNE was associated with better OS and CSS. However, significant differences in OS between black and other races, T1a stage, and radiotherapy were not observed. Significant differences in CSS between black and other races, large cell carcinoma histology, well-differentiated and undifferentiated grades, chemotherapy, and radiotherapy were also not observed (Figure 3). In the subgroup analysis of the scope of LNR, ≥4 LNR had a significantly lower HR OS than 1–3 LNR, with 0 LNR as a reference, regardless of age <70, gender, white race, adenocarcinoma, moderately and poorly differentiated grade, T1b and T1c stage, or chemotherapy and radiotherapy status. Additionally, consistent outcomes for CSS existed in the above subgroups, except for T1c (Figure 4).

## Discussion

Our research demonstrated that more LNE and regions of the LNR were associated with better survival outcomes for patients with stage IA NSCLC treated with lobectomy or bilobectomy. Especially for patients with T1b or T1c stage, more LNE and regions of LNR resulted in significantly better OS and CSS. In a previous study, it was proven that systematic lymph node dissection could improve the identification of occult N2 and was associated with more accurate staging for stage I NSCLC patients, which could guide optimal treatment (14). However, most lymph nodes are presumed to be negative in patients with stage IA NSCLC, which would decrease the therapeutic effect of the more extensive LNE and LNR.

In early-stage NSCLC patients, there is still controversy regarding the management of lymph nodes. In the randomized ACOSOG Z0030 trial, there was no significant survival difference between systematic lymph node dissection and sampling in early-stage NSCLC patients (7). However, Wu et al. demonstrated in another randomized trial that the 5-year survival rate of mediastinal lymph node dissection was significantly better than that of mediastinal lymph node sampling (82.16 vs. 57.49%) in stage I NSCLC patients (15). In a retrospective study including 24,273 stage I NSCLC patients, Varlotto et al. consistently demonstrated that lymphadenectomy compared with no lymphadenectomy and more LNE were associated with significantly better OS and CSS (16). In addition, David et al. performed a retrospective study including 15,195 NSCLC patients and demonstrated a higher number of lymph nodes sampled with better OS and CSS in stage I patients (17). However, previous studies did not further study the difference among the extent of lymph node dissection in patients with stage IA NSCLC.

There are some important results from this study that is worthy of attention. First, most patients had more than 4 LNR (> 75%) and more than 3 LNE (> 85%) assessed, regardless of T stage (Table 1). This reflects the close attention given to the management of lymph nodes in early-stage NSCLC by thoracic surgeons. Second, more than 40% of patients had either 3–8 or ≥9 LNE, which indicated that the number of LNE remains controversial in early-stage NSCLC. At present, the ESMO and NCCN guidelines do not recommend a minimum number of LNE and LNR for patients with stage IA NSCLC (3, 4). According to previous studies, the positive prognostic effects of more extensive dissection have been demonstrated in early-stage colon cancer (18), breast cancer (19), and gastric cancer (20). Additionally, Rucker et al. consistently demonstrated that the assessment of more lymph nodes was associated with better survival in pT1–2N0M0 small cell lung cancer (21). In our study, the lowest HR of OS and CSS existed in both ≥9 LNE and ≥4 LNR via univariate Cox proportional hazards regression analysis. However, ≥9 LNE only had a significant independent effect on CSS, and ≥4 LNR only had a significant independent effect on OS via multiple analyses (Table 2). The correlation between LNE and LNR can explain the outcome of multiple analyses, which demonstrated that the effect of LNE on OS may be caused by the LNR and that the effect of LNR on CSS may be caused by LNE (Tables 3–5). However, a significant effect of different LNE and LNR assessments on survival was not observed among stage IA1 NSCLC patients (Figures 1, 2). Thus, extensive lymph node dissection should be recommended for stage IA2 and IA3 NSCLC patients and is optimal for stage IA1 NSCLC patients. Third, chemotherapy and radiotherapy proved to be associated with significantly worse survival in patients with stage IA NSCLC by the Cox proportional hazards regression model, which was also demonstrated by a previous study (22). However, because of the small number of patients with stage IA NSCLC receiving chemotherapy and radiotherapy, the negative effect of chemotherapy and radiotherapy still needs to be confirmed by randomized controlled trials.

There are some advantages to our study. First, we restaged the patients diagnosed between 2004 and 2015 according to the eighth edition TNM staging, which is recommended in the present guidelines (23). Second, all selected patients with stage IA NSCLC were treated with anatomic pulmonary resection, which is the standard therapy for this group of patients. Thus, the bias associated with different surgical procedures was eliminated. Third, we analyzed the effects of the number of LNE and the scope of LNR on patients with stage IA NSCLC with different T stages, which has not been simultaneously studied in previous studies. Fourth, we included many prognostic factors in the Cox proportional hazards regression model, including age, gender, race, histology, grade, T stage, chemotherapy, and radiotherapy. Ost et al. previously demonstrated that demographic and pathological characteristics were associated with prognostic effects (24). Thus, radiotherapy and chemotherapy were included to further explore the effect of adjuvant therapy on patients with stage IA NSCLC. Fifth, the data of 12,490 patients with stage IA NSCLC were collected from the SEER database rather than from a single institute in order to yield more credible results to guide clinical practice.

However, there are also several limitations to our study. First, as this was a retrospective study, the methods of lymph node dissection were not available, and the chemotherapy and radiotherapy regimens were also unknown. Thus, the results from our study still need to be confirmed in randomized controlled trials. Second, Koike et al. demonstrated that age and tumor size were significant predictors of mediastinal lymph node metastasis in clinical patients with stage IA NSCLC (25). However, the number of LNE in different nodal stations was not identified in our study, which indicated that there probably existed inadequate mediastinal lymph node dissection in patients with stage IA NSCLC. Additionally, the number of LNE in intrapulmonary and mediastinal lymph node stations could respectively have a prognostic effect on patients with stage IA NSCLC, which needs to be further explored.

## Conclusion

This study contributes knowledge with the goal of resolving the existing controversy about extensive lymph nodes dissected in patients with stage IA NSCLC treated with standard curative anatomic pulmonary resection. Especially in the T1b and T1c subgroups, postoperative patients with more extensive lymph nodes dissected had significantly better OS and CSS.

## Data Availability Statement

Publicly available datasets were analyzed in this study. This data can be found at: https://seer.cancer.gov/about/overview.html and the reference number was 14,683-Nov2019.

## Author Contributions

LZ, DZ, and RZ: conception and design. YL, YW, and LZ: administrative support. DZ, RZ, XZ, XY, and KX: provision of study materials or patients. DZ, RZ, GW, ZH, and LY: collection and assembly of data. DZ, RZ, WW, and GG: data analysis and interpretation. All authors wrote the manuscript and approved the final version of the manuscript.

## Conflict of Interest

The authors declare that the research was conducted in the absence of any commercial or financial relationships that could be construed as a potential conflict of interest.

## Publisher's Note

All claims expressed in this article are solely those of the authors and do not necessarily represent those of their affiliated organizations, or those of the publisher, the editors and the reviewers. Any product that may be evaluated in this article, or claim that may be made by its manufacturer, is not guaranteed or endorsed by the publisher.
